# Non-alcoholic fatty liver disease associated with greater herpes zoster risk than alcoholic fatty liver disease

**DOI:** 10.1186/s40001-023-01524-6

**Published:** 2023-12-02

**Authors:** Cheng-Wei Yu, Chia-Hung Chen, Yung-Chi Cheng, Wen-Che Hsieh, Tzu-Ju Hsu, Fuu-Jen Tsai, Chao-Yu Hsu

**Affiliations:** 1 Department of Nutrition, Ditmanson Medical Foundation, Chia-Yi Christian Hospital, Chia-Yi City, 600 Taiwan; 2 Department of Medical Education, Ditmanson Medical Foundation, Chia-Yi Christian Hospital, Chia-Yi City, 600 Taiwan; 3 Department of Medical Imaging, Ditmanson Medical Foundation, Chia-Yi Christian Hospital, Chia-Yi City, 600 Taiwan; 4 Department of Rehabilitation, Ditmanson Medical Foundation Chia-Yi Christian Hospital, Chia-Yi City, 600 Taiwan; 5 Department of Chinese Medicine, Ditmanson Medical Foundation, Chia-Yi Christian Hospital, Chia-Yi City, 600 Taiwan; 6 Management Office for Health Data, Clinical Trial Research Center, China Medical University Hospital, Taichung, 404 Taiwan; 7 School of Chinese Medicine, College of Chinese Medicine, China Medical University, Taichung, 404 Taiwan; 8 Department of Medical Research, China Medical University Hospital, Taichung, 404 Taiwan; 9 Division of Medical Genetics, China Medical University Children’s Hospital, Taichung, 404 Taiwan; 10 Department of Biotechnology and Bioinformatics, Asia University, Taichung, 413 Taiwan; 11 Department of Artificial Intelligence and Healthcare Management, Central Taiwan University of Science and Technology 406, Taichung, Taiwan; 12 Department of Medical Imaging and Radiological Sciences, Central Taiwan University of Science and Technology, Taichung, 406 Taiwan; 13 Center for General Education, National Taichung University of Science and Technology, Taichung, 404 Taiwan; 14 Department of General Education, National Chin-Yi University of Technology, Taichung, 411 Taiwan

**Keywords:** Non-alcoholic fatty liver disease, Alcoholic fatty liver disease, Herpes zoster

## Abstract

**Background:**

Disease-related stress can trigger the occurrence of herpes zoster (HZ). Fatty liver disease (FLD) can have adverse effects on the human body and may induce stress in affected individuals. In this study, we investigated whether FLD is associated with an elevated risk of HZ.

**Methods:**

For this study, we utilized data from the National Health Insurance Research Database, patients with FLD from 2000 to 2017 were observed (follow-up until 2018). Patients were considered to have FLD if they had at least two outpatient visits or at least one admission record with a diagnostic code of FLD. Patients with FLD were matched 1:1 by age, sex, comorbidities, and index year with control patients. Additionally, the FLD was further categorized into non-alcoholic fatty liver disease (NAFLD) and alcoholic fatty liver disease (AFLD) groups. Multivariable Cox proportional hazards model was used to calculate the incidence rate and adjusted hazard ratio (aHR) of HZ for FLD and AFLD and for various age groups, sex and comorbidities. Cumulative incidence curve for HZ was plotted through the Kaplan–Meier method, and p-value was calculated using the log-rank test.

**Results:**

After 1:1 propensity-score matching, each cohort comprised 62,418 patients. The FLD cohort was further divided into NAFLD and AFLD groups, which respectively comprised 55,709 and 6709 patients. The FLD cohort had a risk of HZ significantly higher than that of the control cohort (aHR = 1.06; p < 0.001). Additionally, the NAFLD group exhibited a significantly higher risk of HZ than did the AFLD group (aHR = 1.22; p < 0.001). Among patients without any comorbidities, those with FLD had a higher risk of HZ than did those without FLD (aHR = 1.14; p < 0.001).

**Conclusion:**

Patients with FLD are at an increased risk of HZ development. Additionally, NAFLD is associated with a higher risk of HZ than AFLD. Therefore, patients with NAFLD should be informed of their increased risk of HZ.

## Background

Fatty liver disease (FLD) is caused by the storage of excess fat in the liver. Alcoholic fatty liver disease (AFLD) is caused by heavy drinking. Wong et al. reported that 4.3% of 34,423 respondents to the 2001–2016 National Health and Nutrition Examination Survey had diagnose of AFLD [1]. Non-alcoholic fatty liver disease (NAFLD) is common in individuals with overweight or obesity. The global prevalence of NAFLD in adults is approximately 32%, and the prevalence is higher in men (40%) than in women (26%). The prevalence of NAFLD has increased over time, with studies conducted in 2005 or earlier indicating 26% and those conducted in 2016 or later indicating 38% [2]. A meta-analysis of 237 studies estimated that in Asia, the prevalence of NAFLD was 25.28% between 1999 and 2005, 28.46% between 2006 and 2011, and 33.90% between 2012 and 2017 [3]. Although NAFLD is common among patients with obesity, Ye et al. reported that 40.8% of patients with NAFLD do not have obesity, and 19.2% of patients are lean. They also determined that the incidence of NAFLD among non-obese and lean individuals is 12.1% and 5.1%, respectively, in the general population. The authors suggested that screening for NAFLD not be limited to individuals with obesity [4].

Herpes zoster (HZ) presents as a painful rash caused by reactivation of the varicella-zoster virus (VZV). In an analysis of 69 studies, van Oorschot et al. determined the incidence rate (IR) of HZ was between 5.23 and 10.9 per 1,000 person-years [5]. The risk of HZ recurrence has been reported to be between 1 and 6% [6]. The patients who are immunocompromised have a higher risk of HZ infection. McKay et al. reviewed 34 publications and reported the IR of HZ in the patients with immunocompromised was between 9 and 92 per 1000 person-years. Moreover, the highest incidence of HZ was observed in patients who had undergone hematopoietic cell transplants, followed by those with hematologic malignancies and those with solid organ transplants [7]. Thus, the risk of HZ among patients who are immunocompromised warrants greater attention.

Disease-related stress can trigger the reactivation of VZV. Infectious disease, including those are bacterial [8] or viral [9] and that cause chronic musculoskeletal [10–12] or urogenital [13, 14] pain, have been associated with HZ. FLD can have adverse effects on the human body and may induce stress in affected individuals. In this study, we investigated whether FLD is associated with an elevated risk of HZ.

## Materials and methods

### Data source

The National Health Research Institutes of Taiwan have been promoting the use of the National Health Insurance Research Database (NHIRD) since 2000, which contains the medical records of nearly all Taiwanese people and is a valuable resource for academic research. For this study, we utilized data from the NHIRD, specifically from the year 2000. The disease coding for this study was based on the *International Classification of Diseases, 9th Revision, Clinical Modification (ICD-9-CM) and International Classification of Diseases, 10th Revision, Clinical Modification (ICD-10-CM)*.

### Study population, outcome and comorbidities

In this cohort study, patients with FLD from 2000 to 2017 were observed (follow-up until 2018); the study population comprised individuals aged between 20 and 100 years. Patients were considered to have FLD if they had at least two outpatient visits or at least one admission record with an *ICD-9-CM* diagnostic code of 571.0 or 571.8 or an *ICD-10-CM* diagnostic code of K70.0, K75.81, or K76.0. Additionally, the FLD was further categorized into NAFLD (ICD-9-CM 571.8; ICD-10-CM K75.81, K76.0) and AFLD (ICD-9-CM 571.0; ICD-10-CM K70.0) groups. Patients diagnosed with FLD were meticulously matched in a one-to-one ratio with control individuals, with the matching criteria encompassing age, sex, comorbidities, and the index year. Patients with HZ (ICD-9-CM 053; ICD-10-CM B02) prior to the index date were excluded. Comorbidity refers to the presence of additional medical conditions alongside an index condition throughout an individual's lifetime [15]. In order to mitigate the influence of data selection bias in this study, we have taken into account comorbidity as one of the confounding factors. The relationship between FLD and HZ may be influenced by the presence of multiple concurrent diseases. Among the comorbidities identified in our investigation are diabetes mellitus (DM) (ICD-9-CM: 250; ICD-10-CM: E08-E13), chronic kidney disease (CKD) (ICD-9-CM: 585; ICD-10-CM: N18.4-N18.9), coronary artery disease (CAD) (ICD-9-CM: 410–414; ICD-10-CM: I20-I25), cancer (ICD-9-CM: 140–208; ICD-10-CM: C), obesity (ICD-9-CM: 278, 783.1; ICD-10-CM: E66, E65, E67, E68, R63.5), and depression (ICD-9-CM: 296.2, 296.3, 296.82, 300.4, 309.0, 309.1, 309.28, 311; ICD-10-CM: F32, F33.0-F33.4, F33.9, F34.1, F43.21, F43.23). Furthermore, comorbidities in this study were determined through diagnoses obtained from either a single hospitalization or a minimum of two outpatient care visits.

### Statistical analysis

Categorical variables such as age group, sex, and the presence of various comorbidities are expressed in term of number and frequency, and the chi-square test was employed to detect differences between the FLD and control groups. Continuous variables such as age and follow-up duration are expressed as means and standard deviations, and the t-test was used to detect the differences. Multivariable Cox proportional hazards model was used to calculate the IR and adjusted hazard ratio (aHR) of HZ for FLD and AFLD and for various age groups, sex and comorbidities. Additionally, HZ risk with and without FLD was calculated with adjustment for age, sex, and comorbidities. Cumulative incidence curve for HZ was plotted through the Kaplan–Meier method, and p-value was calculated using the log-rank test. A two-sided p-value < 0.05 indicated statistical significance. SAS software version 9.4 (SAS Institute, Cary, NC) was used for all statistical analyses, and R Studio was employed for the creation of the graph.

## Results

**Fig. 1:**
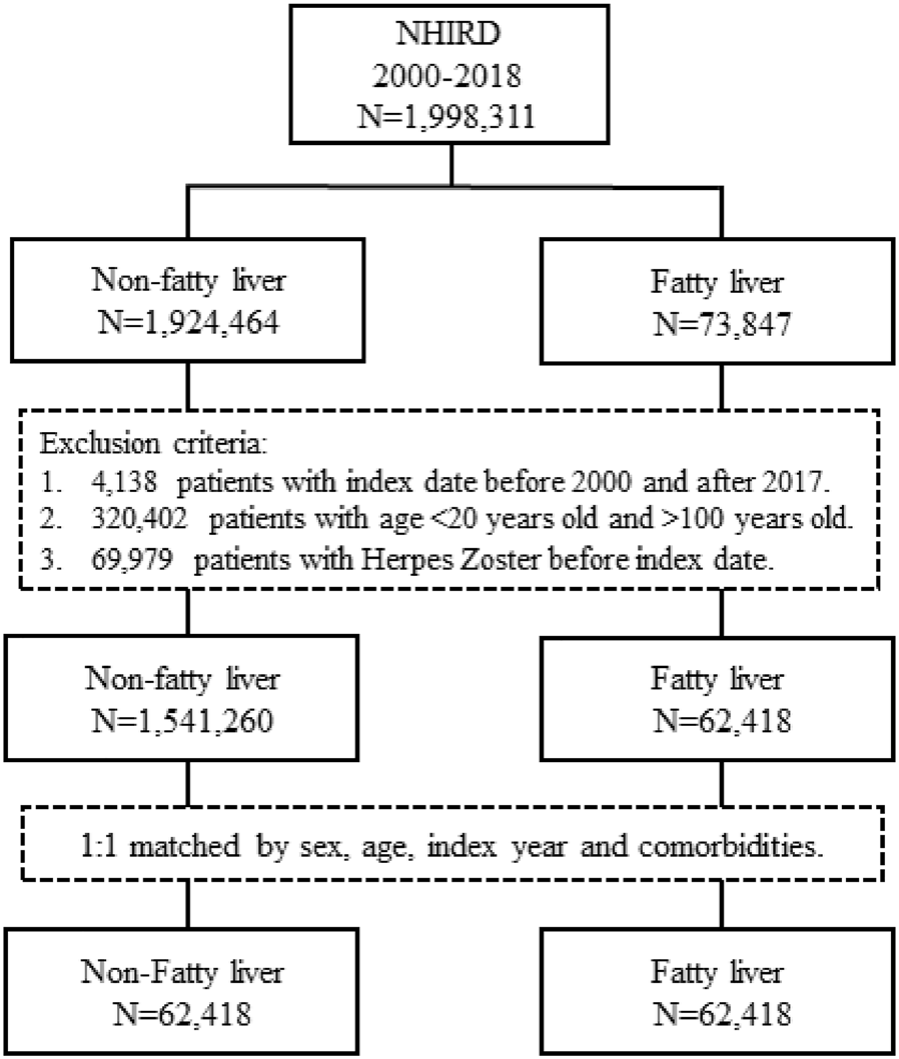
The flow chart of study sample selection from NHIRD Taiwan

Table 1 displays the baseline characteristics of the FLD and control cohorts. After 1:1 propensity-score matching, each cohort comprised 62,418 patients. Figure 1 presents a flow chart illustrating the selection of the study sample from the NHIRD. The distribution of sex, age groups, and comorbidities did not significantly differ. The FLD cohort was further divided into NAFLD and AFLD groups, which respectively comprised 55,709 and 6709 patients.

**Table 1:** Baseline characteristics for individuals with and without fatty liver

Variables	Fatty livermm				*p*-value
	No (N = 62,418)		Yes (N = 62,418)		
	n	%	n	%	
Fatty liver					
Alcoholic fatty liver	-	-	6709	10.75	-
Non-alcoholic fatty liver	-	-	55,709	89.25	-
Sex					
Female	25,721	41.21	25,763	41.27	0.8092
Male	36,697	58.79	36,655	58.73	
Age, years					
20–29	4592	7.36	4641	7.44	0.9924
30–39	10,937	17.52	10,976	17.58	
40–49	14,836	23.77	14,839	23.77	
50–59	15,467	24.78	15,446	24.75	
60–69	9762	15.64	9722	15.58	
> 69	6824	10.93	6794	10.88	
Mean ± SD^a^	50.38	14.63	50.36	14.58	0.7721
Comorbidities					
Diabetes mellitus	15,205	24.36	15,146	24.27	0.6971
Chronic kidney disease	1435	2.30	1458	2.34	0.6653
Coronary artery disease	12,089	19.37	12,042	19.29	0.7362
Cancer	2521	4.04	2544	4.08	0.7414
Obesity	2189	3.51	2279	3.65	0.1703
Depression	6002	9.62	6022	9.65	0.8478
Follow-up time, years					
Mean ± SD^a^	8.49	5.22	8.93	5.24	< 0.001
Median	7.94		8.56		

^a^t-test; SD: standard deviation

**Fig. 2:**
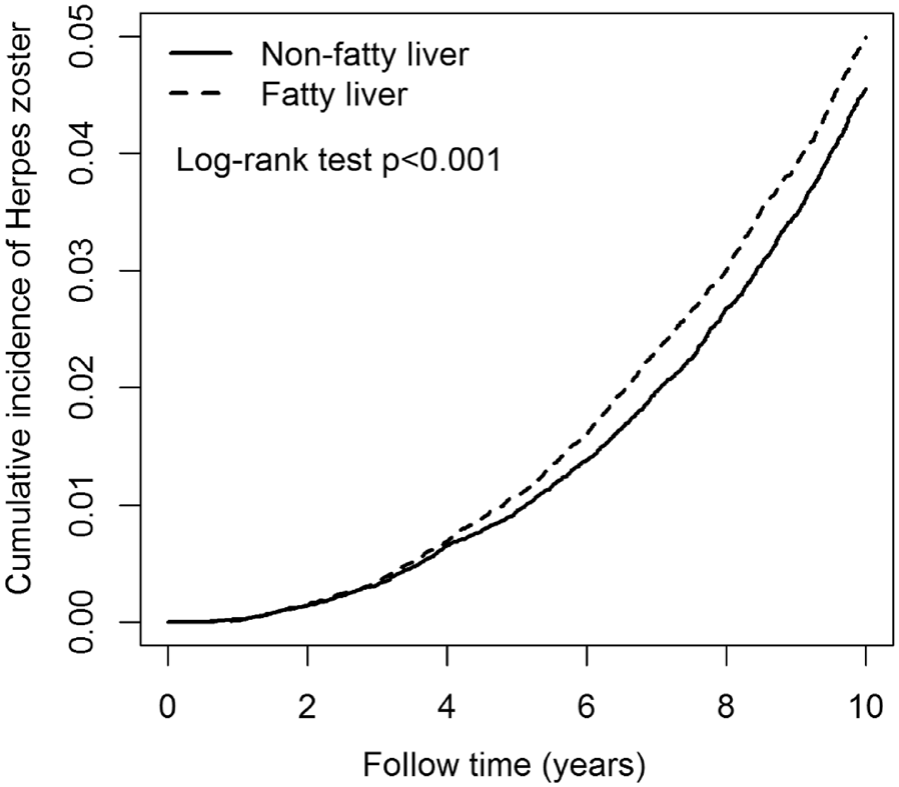
Kaplan–Meier curves of the cumulative incidence rate of herpes zoster during the follow-up period between non-fatty liver group and fatty liver group

**Fig. 3:**
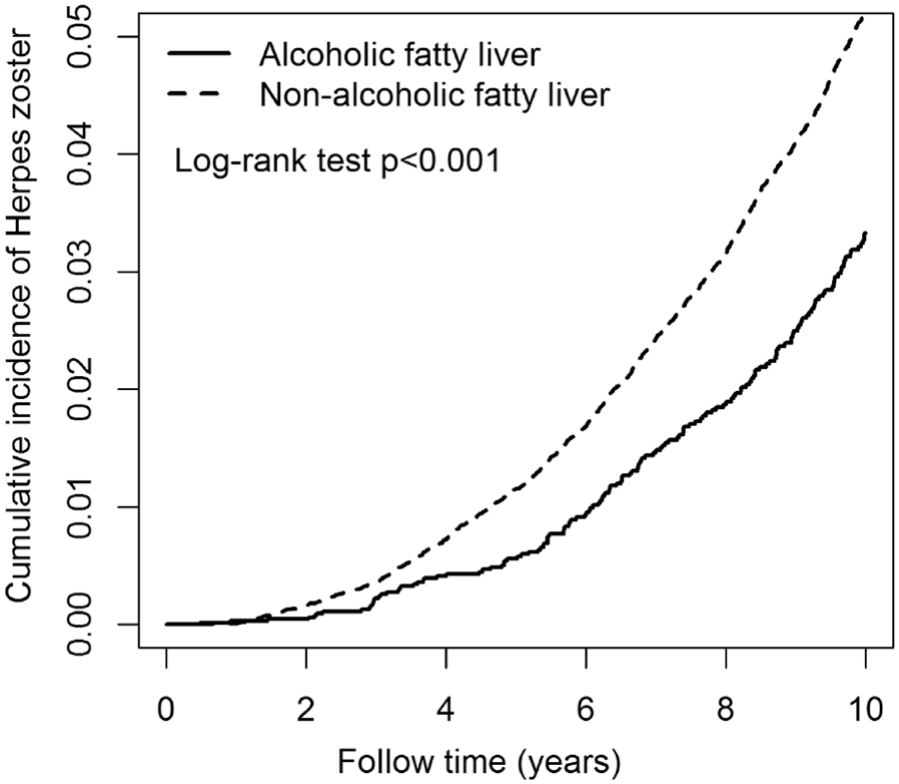
Kaplan–Meier curves of the cumulative incidence rate of herpes zoster during the follow-up period between non- alcoholic fatty liver group and alcoholic fatty liver group

Table 2 presents the events, person-years, IR, and HR for FLD, AFLD, sex, age, and comorbidity groups in relation to HZ risk. The FLD cohort [aHR = 1.06; 95% confidence interval (CI) = 1.02—1.10] had a risk of HZ significantly higher than that of the control cohort. Figure 2 illustrates that the cumulative IR of HZ was significantly higher in the FLD group than in the non-FLD group (p < 0.001, log-rank test). Additionally, the NAFLD group (aHR = 1.22; 95% CI = 1.11—1.33) exhibited a significantly higher risk of HZ than did the AFLD group. Figure 3 presents the Kaplan–Meier plot of the cumulative IR of HZ in the NAFLD and AFLD groups. The cumulative IR of HZ in the NAFLD group was significantly higher than that in the AFLD group (p < 0.001, log-rank test). Men (aHR = 0.94; 95% CI = 0.90—0.98) had a significantly lower risk of HZ than did women. Individuals in the age groups of 30–39 (aHR = 1.15; 95% CI = 1.01—1.31), 40–49 (aHR = 1.81; 95% CI = 1.61—2.05), 50–59 (aHR = 3.11; 95% CI = 2.77—3.51), 60–69 (aHR = 3.83; 95% CI = 3.39—4.32) and > 69 (aHR = 4.91; 95% CI = 4.32—5.59) years had significantly higher risks of HZ than did those in the age group of 20–29 years. Finally, patients with DM (aHR = 1.33; 95% CI = 1.27—1.39), CKD (aHR = 1.57; 95% CI = 1.35—1.82), CAD (aHR = 1.40; 95% CI = 1.33—1.47), cancer (aHR = 1.70; 95% CI = 1.54—1.88), obesity (aHR = 1.69; 95% CI = 1.50—1.91), or depression (aHR = 1.40; 95% CI = 1.31—1.51) had significantly higher risks of HZ than did patients without the respective comorbidities. Table 3 displayed the IR and HR of HZ categorized by age, sex, and comorbidities in AFLD and NAFLD groups. Individuals with AFLD are at an increased risk of developing HZ across various age groups. Those aged 40–49, 50–59, 60–69, and over 69 face significantly higher risks compared to those aged 20–29, with aHR ranging from 2.97 to 7.76. Additionally, having comorbid conditions like DM, CAD, obesity, and depression further elevates the risk of HZ. Similarly, individuals with NAFLD also have a higher risk of HZ in various age groups, with aHR ranging from 1.66 to 4.17 for those aged 40–49, 50–59, 60–69, and over 69 compared to the 20–29 age group. Comorbidities such as DM, CKD, CAD, cancer, obesity, and depression also increase the risk of HZ in individuals with NAFLD.

**Table 2:** Incidences and hazard ratios of herpes zoster for individuals with and without fatty liver by age, sex and comorbidity

Variables	Herpes zoster			cHR	(95% CI)	aHR	(95% CI)
	n	PY	IR				
Fatty liver							
No	4211	530,032	7.94	1.00	(reference)	1.00	(reference)
Yes	5346	557,454	9.59	1.14	(1.09, 1.19)***	1.06	(1.02, 1.10)***
Fatty liver							
Alcoholic fatty liver	506	66,309	7.63	1.00	(reference)	1.00	(reference)
Non-alcoholic fatty liver	4840	491,144	9.85	1.41	(1.29, 1.55)***	1.22	(1.11, 1.33)***
Sex							
Female	4853	449,245	10.80	1.00	(reference)	1.00	(reference)
Male	4704	638,240	7.37	0.68	(0.66, 0.71)***	0.94	(0.90, 0.98)***
Age, year							
20–29	310	90,330	3.43	1.00	(reference)	1.00	(reference)
30–39	828	205,310	4.03	1.16	(1.02, 1.32)*	1.15	(1.01, 1.31)*
40–49	1921	279,967	6.86	1.93	(1.71, 2.18)***	1.81	(1.61, 2.05)***
50–59	3090	269,946	11.45	3.60	(3.20, 4.04)***	3.11	(2.77, 3.51)***
60–69	2147	153,953	13.95	4.77	(4.24, 5.38)***	3.83	(3.39, 4.32)***
> 69	1261	87,978	14.33	6.49	(5.73, 7.35)***	4.91	(4.32, 5.59)***
Comorbidities							
Diabetes mellitus							
No	6955	859,546	8.09	1.00	(reference)	1.00	(reference)
Yes	2602	227,939	11.42	1.92	(1.83, 2.01)***	1.33	(1.27, 1.39)***
Chronic kidney disease							
No	9376	1,072,661	8.74	1.00	(reference)	1.00	(reference)
Yes	181	14,824	12.21	2.57	(2.22, 2.98)***	1.57	(1.35, 1.82)***
Coronary artery disease							
No	7130	902,654	7.90	1.00	(reference)	1.00	(reference)
Yes	2427	184,832	13.13	2.30	(2.19, 2.41)***	1.40	(1.33, 1.47)***
Cancer							
No	9134	1,057,595	8.64	1.00	(reference)	1.00	(reference)
Yes	423	29,890	14.15	2.34	(2.13, 2.59)***	1.70	(1.54, 1.88)***
Obesity							
No	9270	1,055,219	8.78	1.00	(reference)	1.00	(reference)
Yes	287	32,266	8.89	1.48	(1.31, 1.66)***	1.69	(1.50, 1.91)***
Depression							
No	8628	999,558	8.63	1.00	(reference)	1.00	(reference)
Yes	929	87,928	10.57	1.73	(1.62, 1.85)***	1.40	(1.31, 1.51)***

PY: person-year; IR: incidence rate, per 1000 person-years; cHR: crude hazard ratio; aHR: adjusted hazard ratio, adjusted hazard ratio, adjusted for age, sex, index year and comorbidities; CI: confidence interval; *p < 0.05, ***p < 0.001

**Table 3:** Incidences and hazard ratios of herpes zoster by age, sex and comorbidity in alcoholic fatty liver and non-alcoholic fatty liver groups

Variables	Herpes zoster			cHR	(95% CI)	aHR	(95% CI)
	Event	PY	IR				
Alcoholic fatty liver							
Sex							
Female	208	19,111	10.88	1.00	(reference)	1.00	(reference)
Male	298	47,198	6.31	0.62	(0.52, 0.74)***	0.83	(0.69, 1.00)
Age, year							
20–29	11	5090	2.16	1.00	(reference)	1.00	(reference)
30–39	53	15,259	3.47	1.74	(0.91, 3.33)	1.76	(0.92, 3.37)
40–49	132	20,479	6.45	3.15	(1.70, 5.82)***	2.97	(1.60, 5.51)***
50–59	170	15,894	10.70	5.54	(3.01, 10.19)***	4.79	(2.59, 8.85)***
60–69	95	6609	14.37	7.97	(4.27, 14.89)***	6.35	(3.37, 11.99)***
> 69	45	2978	15.11	10.00	(5.19, 19.42)***	7.76	(3.96, 15.22)***
Comorbidities							
Diabetes mellitus							
No	391	55,367	7.06	1.00	(reference)	1.00	(reference)
Yes	115	10,945	10.51	2.12	(1.72, 2.61)***	1.32	(1.06, 1.65)*
Chronic kidney disease							
No	499	65,472	7.62	1.00	(reference)	1.00	(reference)
Yes	7	837	8.36	1.86	(0.88, 3.93)	1.18	(0.56, 2.50)
Coronary artery disease							
No	395	57,494	6.87	1.00	(reference)	1.00	(reference)
Yes	111	8816	12.59	2.58	(2.08, 3.19)***	1.49	(1.19, 1.88)***
Cancer							
No	496	65,300	7.60	1.00	(reference)	1.00	(reference)
Yes	10	1009	9.91	1.87	(1.00, 3.49)	1.37	(0.73, 2.58)
Obesity							
No	487	65,018	7.49	1.00	(reference)	1.00	(reference)
Yes	19	1291	14.71	2.90	(1.83, 4.59)***	3.00	(1.86, 4.83)***
Depression							
No	453	61,521	7.36	1.00	(reference)	1.00	(reference)
Yes	53	4789	11.07	2.13	(1.60, 2.83)***	1.50	(1.12, 2.02)**
Non-alcoholic fatty liver							
Sex							
Female	2488	211,826	11.75	1.00	(reference)	1.00	(reference)
Male	2352	279,318	8.42	0.72	(0.68, 0.77)***	0.96	(0.90, 1.02)
Age, year							
20–29	163	40,238	4.05	1.00	(reference)	1.00	(reference)
30–39	414	87,785	4.72	1.13	(0.94, 1.35)	1.12	(0.93, 1.34)
40–49	914	121,055	7.55	1.76	(1.49, 2.08)***	1.66	(1.40, 1.96)***
50–59	1525	122,712	12.43	3.19	(2.71, 3.75)***	2.79	(2.37, 3.29)***
60–69	1116	74,190	15.04	4.06	(3.44, 4.78)***	3.32	(2.81, 3.94)***
> 69	708	45,165	15.68	5.37	(4.53, 6.37)***	4.17	(3.50, 4.98)***
Comorbidities							
Diabetes mellitus							
No	3486	381,992	9.13	1.00	(reference)	1.00	(reference)
Yes	1354	109,154	12.40	1.77	(1.66, 1.88)***	1.28	(1.20, 1.36)***
Chronic kidney disease							
No	4751	483,715	9.82	1.00	(reference)	1.00	(reference)
Yes	89	7430	11.98	2.10	(1.70, 2.60)***	1.35	(1.10, 1.67)**
Coronary artery disease							
No	3584	403,266	8.89	1.00	(reference)	1.00	(reference)
Yes	1256	87,878	14.29	2.18	(2.04, 2.33)***	1.41	(1.31, 1.51)***
Cancer							
No	4606	475,585	9.68	1.00	(reference)	1.00	(reference)
Yes	234	15,559	15.04	2.02	(1.77, 2.30)***	1.52	(1.33, 1.74)***
Obesity							
No	4703	475,681	9.89	1.00	(reference)	1.00	(reference)
Yes	137	15,463	8.86	1.29	(1.09, 1.54)**	1.53	(1.29, 1.81)***
Depression							
No	4388	450,667	9.74	1.00	(reference)	1.00	(reference)
Yes	452	40,478	11.17	1.62	(1.47, 1.78)***	1.35	(1.22, 1.49)***

PY: person-year; IR: incidence rate, per 1000 person-years; cHR: crude hazard ratio; aHR: adjusted hazard ratio, adjusted hazard ratio, adjusted for age, sex, index year and comorbidities; CI: confidence interval; *p < 0.05, **p < 0.01, ***p < 0.001

Table 4 displays the results for various subgroupings of the FLD cohort. Within the FLD cohort, the risk of HZ was significantly higher for men (IR = 8.12 vs. 6.59; aHR = 1.07; 95% CI = 1.01—1.13), patients aged 30–39 years (IR = 4.53 vs. 3.53; aHR = 1.24; 95% CI = 1.08—1.42), patients aged 40–49 years (IR = 7.39 vs. 6.32; aHR = 1.10; 95% CI = 1.00—1.20), and patients without any comorbidities (IR = 7.93 vs. 6.37; aHR = 1.14; 95% CI = 1.08—1.21), as compared with their counterparts in the control cohort.

**Table 4:** Cox proportional hazards regression analysis for the risk of herpes zoster

Variables	Fatty liver						cHR (95% CI)	aHR (95% CI)
	No			Yes				
	n	PY	IR	n	PY	IR		
Sex								
Female	2157	218,307	9.88	2696	230,937	11.67	1.11 (1.05, 1.17)***	1.06 (1.00, 1.12)
Male	2054	311,724	6.59	2650	326,516	8.12	1.17 (1.10, 1.24)***	1.09 (1.03, 1.15)**
Age, year								
20–29	136	45,003	3.02	174	45,327	3.84	1.25 (1.00, 1.56)	1.24 (0.99, 1.55)
30–39	361	102,266	3.53	467	103,044	4.53	1.26 (1.10, 1.44)**	1.24 (1.08, 1.42)**
40–49	875	138,433	6.32	1046	141,534	7.39	1.12 (1.02, 1.22)*	1.10 (1.00, 1.20)*
50–59	1395	131,340	10.62	1695	138,606	12.23	1.07 (0.99, 1.15)	1.05 (0.98, 1.13)
60–69	936	73,154	12.79	1211	80,799	14.99	1.03 (0.95, 1.12)	1.01 (0.93, 1.10)
> 69	508	39,835	12.75	753	48,144	15.64	0.98 (0.88, 1.10)	0.97 (0.86, 1.08)
Comorbidities								
No	2088	327,795	6.37	2661	335,404	7.93	1.18 (1.12, 1.25)***	1.14 (1.08, 1.21)***
Yes	2123	202,236	10.50	2685	222,049	12.09	1.03 (0.98, 1.09)	0.98 (0.93, 1.04)

^a^Individuals with any comorbidity of diabetes mellitus, chronic kidney disease, coronary artery disease, cancer, obesity and depression were classified into the comorbidity group; PY: person-year; IR: incidence rate, per 1000 person-years; cHR: crude hazard ratio; aHR: adjusted hazard ratio, adjusted for age, sex, index year and comorbidities; CI: confidence interval; *p < 0.05, **p < 0.01, ***p < 0.001

## Discussion

We observed that patients with FLD had an increased risk of HZ development. In addition, NAFLD is associated with a higher risk of HZ than AFLD. Alcohol consumption exerts an impact on both innate and adaptive immunity in humans. Persistent heavy drinking is linked to a lower count of lymphocytes and an elevated susceptibility to bacterial and viral infections. In contrast, moderate alcohol consumption is associated with decreased inflammation and enhanced vaccination responses [16]. Schmidt et al. [17] discovered that in comparison to low-risk alcohol consumption, neither intermediate-risk nor high-risk consumption showed a higher risk of HZ. Furthermore, there was no elevated relative risk among respondents who reported weekly binge drinking compared to those who did not. Both pathogen-associated molecular patterns and damage-associated molecular patterns are pivotal in activating innate immunity in response to stressors like alcohol. Chronic alcohol consumption increases liver exposure to these molecules, activating macrophages and boosting neutrophil recruitment. In alcoholic liver disease, neutrophil infiltration correlates with a more favorable prognosis, suggesting their dual role in injury and repair [18]. In addition, Barr et al. [16] demonstrate that moderate alcohol consumption increased the production of T cell cytokines such as IL-2, IL-4, IL-10, and IFN-γ, and it also elevated the frequency of lymphocytes. These factors could contribute to the reduced risk of HZ in the AFLD group when compared to the NAFLD group.

Most of our patients (nearly 90%) had NAFLD. Therefore, our discussion focuses on NAFLD. A meta-analysis of 24 studies involving 35,599 patients discovered the prevalence of NAFLD in patients with DM to be 59.67% [19]. In a meta-analysis of 33 publications, Mantovani et al. discovered that compared with patients without NAFLD, patients with NAFLD had a higher risk of DM (HR = 2.19) [20]. Because NAFLD is common in patients with DM, Stefan and Cusi suggested that the management of NAFLD be integrated into the disease treatment plan for patients with DM. The authors also suggested the risk of diabetic complications be stratified on the basis of NAFLD [21]. After a 10-year nested case–control study of 1428 patients, Chuanchaiyakul et al. reported that the IR of HZ was 3.96 per 1,000 person-years [22]. Patients with DM were at a higher risk of HZ than were the patients without DM. Hung et al. analyzed 16 studies and discovered that patients with DM had a higher risk of HZ than did the general population, the relative risk was 1.38. The authors thus suggested that the HZ vaccine be considered for patients with DM [23].

NAFLD increases the incidence of CKD. One study demonstrated that the odds of CKD were significantly higher in patients with NAFLD than in controls (OR = 1.95) [24]. Le et al. analyzed data from 1999–2016 National Health and Nutrition Examination Survey and reported that the prevalence of NAFLD with renal insufficiency increased significantly from 5.7% in 1999–2000 to 7.7% in 2015–2016. Moreover, among patients with NAFLD, the patients with severe renal insufficiency had the highest mortality incidence for all-cause mortality (104.1 per 1,000 person-years), compared with other causes of mortality (50.88 per 1,000 person-years) [25]. Han discovered the mechanism by which NAFLD leads to kidney disease: NAFLD-induced insulin resistance aggravates systemic chronic inflammation and oxidative stress, leading to extrahepatic organ dysfunction [26]. Lai et al. observed that the IR of HZ was higher in patients with predialysis CKD (8.76 per 1000 person-years) than in those without CKD (6.27 per 1,000 person-years). The patients with predialysis CKD had a 1.4-fold risk of HZ development [27]. Therefore, HZ vaccine may also be recommended for patients with CKD [28].

Ng et al. reported a risk of depression for individuals with NAFLD was 12% higher than that for those without NAFLD [29]. In another study, patients with NAFLD had a significantly higher risk of depression (OR = 1.13) than did those without NAFLD, and patients with depression had a significantly higher risk of developing NAFLD (OR = 1.46) than did those without. The authors demonstrated that NAFLD and depression are highly correlated and can interact [30]. Because concurrent depression in NAFLD can amplify the likelihood of negative health consequences, Ng et al. suggested that early screening for depression in high-risk individuals with NAFLD can improve their well-being [29]. Two population-based studies have demonstrated strong associations between depression and HZ, indicating that the HR of depression for HZ development is 1.1 (compared with no depression) [31, 32]. Therefore, NAFLD, depression, and HZ are likely to be interrelated.

In addition to DM, CKD, and depression, our analysis revealed that CAD, cancer, and obesity were associated with a higher risk of HZ compared to individuals without these conditions (Table 2). Nevertheless, within the subset of patients who did not have the mentioned comorbidities, those with FLD displayed a significantly elevated risk of HZ when compared to their counterparts without FLD (Table 4). Additionally, within the cohort of individuals afflicted with AFLD, the augmented HR predominantly stem from the advancing age of the subjects, although there exist sporadic instances of non-significant outcomes for specific comorbid conditions. Conversely, when examining the NAFLD group, all encountered comorbidities exert a statistically significant impact on the susceptibility to HZ (Table 3). These observations suggest a mutually reinforcing relationship between NAFLD and various comorbidities in augmenting the risk of HZ. This finding implies that FLD could be a significant factor in precipitating the onset of HZ, especially in cases of NAFLD. Consequently, the health impact associated with FLD should not be underestimated or overlooked.

This study has some limitations. The NHIRD only contains information related to health insurance benefits, and thus does not include patients’ data such as those on disease severity, lifestyle (such as smoking, alcohol consumption), or self-funded medical treatment. Thus, our research is potentially subject to bias. In addition, the quality and reliability of the data may be limited by differences among medical institutions, variations in medical behavior, and incomplete data collection. However, the NHI Administration has a rigorous review system, and severe penalties are imposed for improper diagnoses and prescriptions. Therefore, the results of our empirical analysis of big data from the NHIRD can be used by clinicians as a reference.

## Conclusion

Patients with FLD are at an increased risk of HZ development. Additionally, NAFLD is associated with a higher risk of HZ than AFLD. Therefore, patients with NAFLD should be informed of their increased risk of HZ.

## Data Availability

The data used in this study were sourced from the National Health Insurance Research Database, Taiwan. According to the Personal Data Protection Act, data cannot be made public. Researchers may request access to the data for research purposes through the Taiwan National Health Insurance Administration's website (http://nhird.nhri.org.tw).

## Abbreviations

AFLD: Alcoholic fatty liver disease
aHR: Adjusted hazard ratio
CAD: Coronary artery disease
CI: Confidence interval
CKD: Chronic kidney disease
DM: Diabetes mellitus
FLD: Fatty liver disease
HZ: Herpes zoster
IR: Incidence rate
NAFLD: Non- Alcoholic fatty liver disease
NHIRD: National Health Insurance Research Database
VZV: Varicella-zoster virus
